# Medical students’ readiness and perceptions about Interprofessional Education: A cross sectional study

**DOI:** 10.12669/pjms.36.4.2214

**Published:** 2020

**Authors:** Hana Alzamil, Sultan Ayoub Meo

**Affiliations:** 1 Dr. Hana Alzamil, MBBS, Ph.D. Department of Physiology, College of Medicine, King Saud University, Riyadh, Saudi Arabia; 2 Prof. Sultan Ayoub Meo, MBBS, Ph.D. Department of Physiology, College of Medicine, King Saud University, Riyadh, Saudi Arabia

**Keywords:** Collaboration, Communication skills, Interprofessional Education, Medical education

## Abstract

**Objectives:**

Interprofessional Education (IPE) provides an environment where learners demonstrate the knowledge, skills and attitude required to manage the complex clinical scenarios in a collaborative and interprofessional manner. The actual sphere of influence of Interprofessional Education in many medical schools has been limited. Therefore, the present study aim was to evaluate the medical students’ readiness and perception of Interprofessional Education in a medical college in Saudi Arabia.

**Methods:**

This questionnaire based cross sectional study was executed in the Department of Physiology, College of Medicine, King Saud University during the period September 2016 to December 2017, using the 19-item Readiness for Interprofessional Learning Scale (RIPLS) with four subscales teamwork and collaboration, negative professional identity, positive professional identity and roles and responsibilities. The questionnaire was e-mailed to 1411 medical students and responses were analyzed using 5-point Likert scale.

**Results:**

A total of 158 medical students and trainees responded to the survey, 69 (43.6%) were males and 89 (56.4%) were females. The majority of participants 122-148 (77-94%) acknowledged the positive impact of IPE on teamwork and collaboration, more than two thirds 105 (64.45%) disagreed with negative attitude and 110-126 (70-80%) showed positive professional identity.

**Conclusions:**

Medical students showed a positive perception and ready to adopt the Interprofessional Educational allied activities in medical schools. The shared academic events would improve in clarifying the roles and responsibilities of medical students in health care professions.

## INTRODUCTION

Interprofessional Education (IPE) is essential to prepare the learners in healthcare professions to become effective members, provide better healthcare services and meet professional and ethical standards. IPE provides an environment where learners are able to demonstrate the knowledge, skills and attitude required to deal with complex clinical scenarios in a collaborative and interprofessional manner. IPE helps in minimizing the learning gaps for successful professional career.^1^ It provides learning process by which students with numerous cultural contexts learn together with interaction as this is important “to collaborate in providing promotive, preventive, curative, rehabilitative, and other health-related services”.^2^ Many healthcare systems are trying to meet the health and wellbeing needs of community due to disintegration and lack of collaboration in practice. The poor communication among healthcare professionals has been linked with poor patient outcomes.^3^

Interprofessional Education is mandatory in many health care professional courses and is integrated in the undergraduate curriculla.^4^ It requires the development of innovative intellectual ambiance through social interaction and collaboration. The learning requires an acknowledgement and appreciation of the characteristic socialization and creation of learning opportunities with positive interaction.^5^ IPE allows the learners from different disciplines to learn collaborative behaviors.^6^ In health care sectors, establishing interdisciplinary team training programs involving simulations,^5^ team building as well as communication skills^7^ among members is highly recommended to improve the Interprofessional Collaboration (IPC). IPE is essential in preparing the learners in all healthcare professions to become effective members of collaborative teams who complement and complete the care provided.^8^ Medical students need to develop high standards of knowledge, skills and professional attitude to manage the complex clinical scenarios in a collaborative, interprofessional manner.^9^ The learners learn integration, collaborative competencies,^10^ respect and professional responsibilities which eventually enhance the patient care.^11^ The actual sphere of influence of Interprofessional Education in many medical schools has been limited. Therefore, the present study aimed to evaluate the medical students’ readiness and perceptions about IPE in a medical institution in Saudi Arabia.

## METHODS

### Study design and settings

This cross-sectional study was performed in the “Department of Physiology, College of Medicine, King Saud University” from September 2016 to December 2017.

### Study participants

The study was conducted by using a validated questionnaire (Readiness for Interprofessional Learning Scale [RIPLS].^12^ The targeted study population was undergraduate and postgraduate male and female medical students and trainees, College of Medicine and allied university hospitals of King Saud University. The students contact information was obtained from the admission and medical education departments. The questionnaire was e-mailed to 1411 medical students, 819 (58.04%) were males and 592 (41.95%) were females. The e-mail was followed by three more reminders every other week to enhance the response rate. The survey was conducted in English which is the official medium of instruction language at the medical school. Participation in the study was voluntary and anonymous; the purpose of the study was explained in the first page of the questionnaire. The data were collected through an electronic, self-administered questionnaire which included demographic information age, gender, year of study and previous.

### Instrument

The “Readiness for Interprofessional Learning Scale (RIPLS)” was used in its original version that was developed by Parsell and Bligh.^12^ The RIPLS enables the students to reflect on various aspects of Interprofessional Education, and was used to measure student readiness or beliefs about IPE. Parsell and Bligh^12^ familiarized the notion of “Readiness for Interprofessional Learning” as a grading in which students willing to participate in IPE, by using four dimensions including, knowledge and skills for teamwork, roles and responsibilities of self and others, benefits to patients, practice and personal growth, and values. The questionnaire comprises 19 items covering four subscales: “teamwork and collaboration, negative professional identity, positive professional identity, and roles and responsibilities”. The study participants indicate their level of agreement on a 5-point Likert scale, “strongly disagree, disagree, neutral, agree, or strongly agree”. The questionnaire explores the students’ perceptions towards Interprofessional Learning. The questionnaire was pre-validated in various academic cultural contexts.^13^,^14^

### Ethics Statement

The study protocol was reviewed and approved by the “Ethical Review Board, Department of Family and Community Medicine, College of Medicine, King Saud University, Riyadh, KSA” (Ref No. CMED305-MB8-2013-14, dated December 8, 2019).

### Statistical analysis

Data were analyzed using Statistical Package for Social Sciences (SPSS) version 23 (SPSS Inc., IBM-SPSS, Chicago, Illinois, USA). Results are presented in percentages. The prevalence of awareness was determined by comparing outcome measures. Numerical variables were reported as the mean ± standard deviation. P<0.05 was considered significant.

## RESULTS

**Fig.1:**
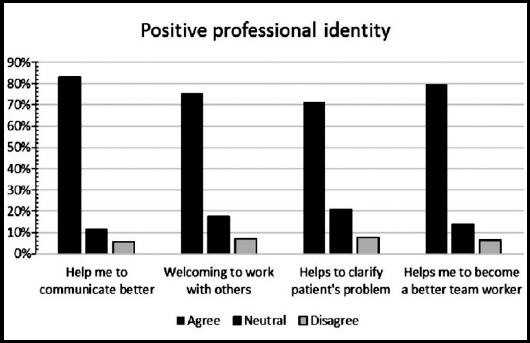
Positive professional identity among medical students.

**Fig.2:**
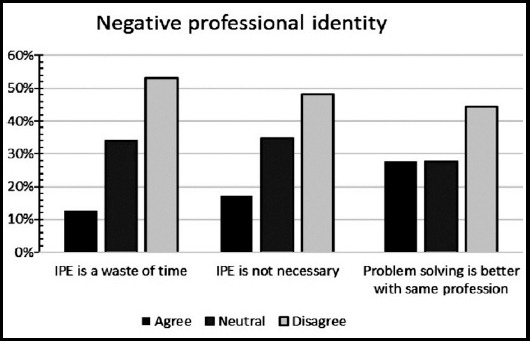
Negative professional identity among medical students.

**Fig.3:**
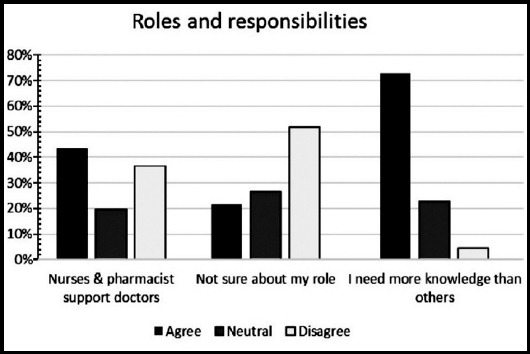
Identification of role and responsibilities among medical students.

**Fig.4:**
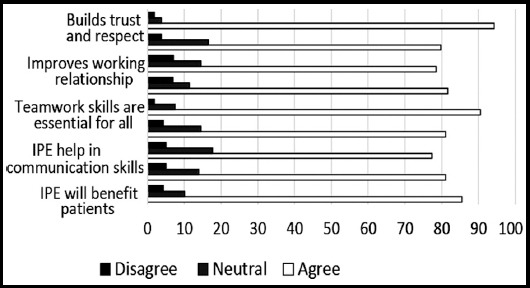
Identification of teamwork and collaboration among medical students.

A total of 158 medical trainees responded to the survey, yielding a response rate of 11.19%. Sixty-nine (43.6%) were males and 89 (56.3%) were females. Forty-two percent of participants were from preclinical years (20.3% first years, 21.5% second years’ students), 44% were from clinical years (14.6% third years, 17.1% fourth years, 13.9% final year students), and 12.7% were interns and trainees. Thirty-seven students (23.4%) had a previous experience with Interprofessional Education. The perception of students regarding the importance of IPE in improving collaboration and teamwork skills is shown in (Fig.1). High percentage of participating medical students, ranged between 77% and 94%, valued the positive impact of IPE on teamwork and collaboration (Fig.1). Low percentage of participants (ranged between12% and 27%) agreed with statements describing negative professional identity (Fig.1 and 2). Approximately 80% of respondents have positive professional identity. In the response to roles and responsibilities subscale, more than 40% of participating students were not sure about their roles and responsibilities and were uncertain about the role of nurses and pharmacists in the health care team (Fig.3 and 4).

## DISCUSSION

Interprofessional Education, gathering different professional groups into carefully facilitated learning opportunities improve their approaches, manage their negative stereotyping, prejudice and strengthen their collaboration.^12^,^13^ In this study, we found that medical students at King Saud University highly valued IPE as a tool to improve their communication skills, practice teamwork competencies, build mutual trust and respect, improve patient care, and facilitate problem solving skills. Our findings are consistent with the results of previous studies conducted in the Gulf region and Saudi Arabia.^14^-^17^

A previous study involving the medicine, nursing, pharmacy and applied medical science students reported an agreement among the students, that collaboration and working as a team with other health care professionals is important.^16^ Similarly, medical students from King Abdulaziz University valued IPE and expected that patient care as well as satisfaction of health care providers will improve after implementation of IPE in their education.^15^ A recent study conducted in Saudi Arabia showed that students in various health care professions have a positive attitude and were prepared for shared learning.^17^ Another study concluded that although medical students were well prepared for IPE, health profession students appreciate the opportunity offered by IPE to hone their leadership and communication skills and to clarify professional roles and boundaries.^18^ A prospective controlled study reported that a short IPE intervention program not only significantly improved students’ attitudes towards IP learning but also improved self-reported confidence and effectiveness as a member in the health care team.^19^

Our medical students in the current study had low scores in their response to the negative professional identity while their scores were very high in response to positive professional identity. The finding showed that the students are ready to share their learning with peer students from wide-ranging health professions, as they showed interest in working in multi-professional teams to improve their communication skills and to provide better health care. In line with our findings, Al-Eisa et al.^16^, reported that all students from different health professions valued the opportunity to share their learning and experience with other students from different disciplines.^16^ Interestingly, AlQahtani et al.^17^ observed that senior students have positive attitudes toward Interprofessional learning than junior students.

Similar to the findings of a recent study, our study showed that approximately half of the participants were unsure about their professional role and only a third of them disagreed with the idea that nurses and pharmacists are there to support physicians.^20^ These findings might be due to the high percentage of preclinical students among the participants in our study which may reflect their lack of clinical experience. Wilby et al.^21^, reported that nutrition and pharmacy students became more mindful of their professional roles in the third and fourth years of their study.^21^ Participation in healthcare experience as well as being at an advanced learning level were associated with more positive attitude towards IPE.^22^ McFadyen et al. and colleagues^23^ argued that lack of professional experience in early undergraduate students could be the reason for weak values of the roles and responsibilities sub-scale. In line with our observation, Horsburgh et al. and coworkers^24^ reported that compared to pharmacy and nursing students, medical students were the least sure about their roles and responsibilities. On the contrary, Mahler et al.^25^, observed that graduates were less certain than undergraduates about essence of their professional role and responsibility. Interestingly, both McFadyen et al.^23^ and Horsburgh and coworkers^24^ identified that the internal consistency of the RIPLS suggests that “roles and responsibilities” sub-scale was unreliable.^23^,^24^ Similarly, Mahler et al.^26^, concluded that although in the English version of RIPLS, the internal consistency (Cronbach’s alpha) of the overall scale seems to be satisfactory, the “roles and responsibilities” subscale was unstable and unsatisfactory which has forced some researchers to neglect this scale in their data analysis. A poor information about the roles of diverse health professionals was considered as a barrier to teamwork.

A study that was conducted to analyze the sources of physician-nurse conflict observed that medical and nursing students neither study together nor their curricula provided enough information about the contributions of other health care professions. A study investigating the views of the third and fourth year medical students about the role of nurses showed difficulty among them in defining the nurse’s role relative to their own roles and the majority expressed confusion in defining the interface between nursing and medicine.^27^ One of the findings in the current study shows that more than 70% of participants believed that physicians need to have more knowledge than other health care professionals. Similarly, Horsburgh et al.^24^, reported that medical students believed that they need to obtain more knowledge and skills than nursing or pharmacy students. The same study showed that medical students have the tendency to view doctors as having dominance over other health professionals, which indicate the need for IPE in order to change this attitude and to allow collaborative model to work.^24^ There is an increasing demands for establishing medical education departments in medical schools,^28^ and Interprofessional Education is essential to prepare better health care providers to meet the higher professional and ethical standards.

### Limitations of the study

It includes small sample size, and students from other health care professions were not involved to compare their attitudes with that of medical students. In addition, we were unable to compare the attitude and perception of students at different levels and between genders due to the low response rate.

## CONCLUSIONS

Medical students revealed readiness to contribute the learning activities with their peer students. The findings support the notion that implementation of Interprofessional Education activities will improve the teamwork competencies, communication skills and have a positive impact on medical students’ perception of their roles and responsibilities.

### Author’s Contributions

**HA** presented the original idea, data collection and manuscript writing.

**SAM** literature review, data analysis, final manuscript writing. Authors read and approved the manuscript, and are responsible for the integrity of the work.
